# Effects of robot therapy on upper body kinematics and arm function in persons post stroke: a pilot randomized controlled trial

**DOI:** 10.1186/s12984-020-0646-1

**Published:** 2020-01-30

**Authors:** Ilaria Carpinella, Tiziana Lencioni, Thomas Bowman, Rita Bertoni, Andrea Turolla, Maurizio Ferrarin, Johanna Jonsdottir

**Affiliations:** 1 IRCCS Fondazione Don Carlo Gnocchi, via Capecelatro 66, 20148 Milan, Italy; 2 Movement Neuroscience Research Group, IRCCS San Camillo Hospital, Via Alberoni 70, 30126 Venezia, Lido Italy

**Keywords:** Stroke, Robot therapy, Upper limb, Trunk, Kinematic analysis, Motor strategies

## Abstract

**Background:**

Robot-based rehabilitation for persons post-stroke may improve arm function and daily-life activities as measured by clinical scales, but its effects on motor strategies during functional tasks are still poorly investigated. This study aimed at assessing the effects of robot-therapy versus arm-specific physiotherapy in persons post-stroke on motor strategies derived from upper body instrumented kinematic analysis, and on arm function measured by clinical scales.

**Methods:**

Forty persons in the sub-acute and chronic stage post-stroke were recruited. This sample included all those subjects, enrolled in a larger bi-center study, who underwent instrumented kinematic analysis and who were randomized in Center 2 into Robot (R_Group) and Control Group (C_Group). R_Group received robot-assisted training. C_Group received arm-specific treatment delivered by a physiotherapist. Pre- and post-training assessment included clinical scales and instrumented kinematic analysis of arm and trunk during a virtual untrained task simulating the transport of an object onto a shelf. Instrumented outcomes included shoulder/elbow coordination, elbow extension and trunk sagittal compensation. Clinical outcomes included Fugl-Meyer Motor Assessment of Upper Extremity (FM-UE), modified Ashworth Scale (MAS) and Functional Independence Measure (FIM).

**Results:**

R_Group showed larger post-training improvements of shoulder/elbow coordination (Cohen’s d = − 0.81, *p* = 0.019), elbow extension (Cohen’s d = − 0.71, *p* = 0.038), and trunk movement (Cohen’s d = − 1.12, *p* = 0.002). Both groups showed comparable improvements in clinical scales, except proximal muscles MAS that decreased more in R_Group (Cohen’s d = − 0.83, *p* = 0.018). Ancillary analyses on chronic subjects confirmed these results and revealed larger improvements after robot-therapy in the proximal portion of FM-UE (Cohen’s d = 1.16, *p* = 0.019).

**Conclusions:**

Robot-assisted rehabilitation was as effective as arm-specific physiotherapy in reducing arm impairment (FM-UE) in persons post-stroke, but it was more effective in improving motor control strategies adopted during an untrained task involving vertical movements not practiced during training. Specifically, robot therapy induced larger improvements of shoulder/elbow coordination and greater reduction of abnormal trunk sagittal movements. The beneficial effects of robot therapy seemed more pronounced in chronic subjects. Future studies on a larger sample should be performed to corroborate present findings.

**Trial registration:**

www.ClinicalTrials.gov NCT03530358. Registered 21 May 2018. Retrospectively registered.

## Background

Stroke is a primary cause of long-term disability worldwide [1] with nearly 1.1 million persons in Europe suffering a stroke each year [2]. Importantly, this number is expected to increase to more than 1.5 million cases per year in 2025, mainly due to an aging population [3].

Approximately 70–85% of persons post-stroke present with impairment of an upper limb [4, 5] that persists even after 3–6 months from stroke [6], leading to a significant reduction of independence and quality of life [7]. Consequently, improving upper limb functionality is a core element of stroke rehabilitation to reduce disability and increase the capacity to perform the activities of daily living (ADLs) [8]. Different rehabilitative approaches have been proposed [9, 10], including constraint induced movement therapy [11], functional electrical stimulation [12, 13], virtual reality [14, 15] and robot therapy [16, 17]. Regarding the latter approach, two recent reviews [16, 17] indicated that robot-based rehabilitation is effective in improving ADLs, arm function and muscle strength in persons post-stroke. Previous studies suggested that the advantage of robotic devices, when compared with other physiotherapy approaches, may be the capability of these systems to provide rehabilitation paradigms enabling a strict application of some motor learning principles [18–20] indispensible to promote neural plasticity and reorganization [21–23]. In particular these principles include (1) the provision of highly intensive training involving a large number of goal-directed movements (e.g. center-out reaching of peripheral targets aimed at improving the coordination between shoulder and elbow) [21, 24], (2) the promotion of active participation by the person, also when severely impaired [25], and (3) the provision of real-time sensory feedback (visual and haptic) and quantitative summary feedback that can be used by the participant to correct his/her movement [14, 26]. Importantly, as previously discussed [27, 28], further investigation is needed to evaluate if the application of these motor learning principles can enhance the transfer of the rehabilitation effects also to non-trained tasks and contexts typical of ADLs.

The effects of motor rehabilitation on upper limb function are commonly assessed with clinical scales [29] that are mainly focused on task accomplishment, but do not give quantitative, objective and sensitive information on underlying changes in neuromotor control strategies involving inter-joint coordination and/or compensatory movements [30–33]. As discussed by Levin et al. [30], the main goal of motor rehabilitation is to lead the person to accomplish a task. However, also the assessment of *how* the task is performed is of paramount importance to evaluate whether the person has regain the ability to execute the task with a more physiological upper limb motor pattern (recovery), or he/she has developed compensatory strategies, such as abnormal trunk rotations (compensation) [30, 31, 34–37]. Instrumented motion analysis may provide this information and complement clinical assessment [31–33, 38, 39].

Instrumented analysis is usually performed using quantitative robot-based indexes describing a number of trained and non-trained tasks [28, 40–44]. As summarized in a review by Nordin et al. [45], the most common robot-based parameters describing upper limb movement and sensation include the amplitude of robot-generated forces [40, 41], temporal and speed metrics [40, 43, 44, 46, 47], response latency [46, 47], accuracy indexes [40, 43, 44, 46, 47], path length and range of motion [41, 42, 46, 47], and movement smoothness [40–44, 46, 47]. The test-retest reliability, the discriminant ability and the concurrent validity of these robot-based indexes have been analyzed in a large number of studies. Among these studies, those including the largest samples of persons post-stroke [41, 46–49] found good to excellent reliability [41, 48], good discriminant ability [41, 47], and moderate to high concurrent validity with clinical scales [41, 46, 47, 49]. The main advantage of the robot-based indexes is that they can be easily obtained during the course of the robotic training, thus providing indications about the gradual progression of the participants’ performance [50]. By contrast, the main drawback is that these parameters mainly describe the trajectory of the end-effector during planar tasks executed within the robot workspace that is different from the typical daily living contexts.

This drawback may be partly overcome by using more sophisticated kinematic analysis techniques [32, 33, 38, 51–57] aimed at characterizing the execution of more ecological activities performed outside the robot workspace, including pointing tasks [34, 37] or reaching forward and touching real objects placed on a table, such as boxes [54, 55], cups [51], glasses [32, 33, 57], discs [55], cones [36] and desk bells [52, 53, 56]. Compared to the robot-based indexes, these analyses may provide a more detailed characterization of the different components of a task (e.g. upper limb and trunk movements), thus adding information about the way a task is performed before and after a rehabilitation treatment. This, in turn, may help in assessing the effects of such treatment in terms of neuromotor recovery and/or compensation [30, 34, 37, 50]. However, with the exception of Cirstea and Levin [37] who described trunk and arm motion during a 3D pointing tasks, all the above mentioned studies analyzed activities that mainly involved movements in the horizontal plane, with a minimal vertical component against gravity that is, however, a fundamental aspect of ADLs.

Following these considerations, this pilot study had two aims. The first aim was to assess the effects of planar robotic rehabilitation versus arm-specific physiotherapy in persons post-stroke on motor strategies derived from instrumented kinematic analysis of upper limb and trunk during the execution of a non-trained task involving horizontal and vertical arm movements. The second aim was to compare the effects of the two rehabilitation approaches on arm function as measured by clinical scales. We hypothesized that robot therapy provides larger improvements in the coordination between shoulder and elbow joints and in upper limb impairment, since it enables a rigorous application of the motor learning principles described above, in particular administration of high intensity goal-directed training, promotion of active participation, and provision of feedback.

## Methods

### Study design

This study is part of a larger bi-center randomized controlled trial (the MOSE study, ClinicalTrial.gov, NCT03530358) aimed at testing the efficacy of two technology-based approaches for upper limb rehabilitation in persons post-stroke: (1) virtual reality-based training, administered at IRCCS San Camillo Hospital, Venice, Italy (Center 1), and (2) robot-assisted therapy, administered at IRCCS Don C. Gnocchi Foundation, Milan, Italy (Center 2). The study was retrospectively registered due to coordination issues between centers.

In both centers the study design consisted of a single-blind two-arm randomized 1:1 controlled trial.

Specifically, in the present study we compared the effects of robot therapy (experimental intervention) and arm-specific physiotherapy (control intervention) on upper body kinematics and arm function in all participants post-stroke recruited and randomized in Center 2.

### Participants

A consecutive sample of 116 adults post-stroke from the Neurorehabilitation Department of IRCCS Don Carlo Gnocchi Foundation (Milan, Italy) was assessed for eligibility from March 2015 to November 2017. Inclusion criteria were: first ischemic or hemorrhagic stroke, a score between 1 and 3 at the upper limb sub-item on the Italian version of the National Institute of Health stroke scale (IT - NIHSS) [58], a score higher than 6 out of 66 points on the Fugl-Meyer Motor Assessment of Upper Extremity (FM-UE) scale [59].

Exclusion criteria were: presence of a moderate cognitive decline defined as a Mini Mental State Examination [60] score < 20 points, evidence of severe verbal comprehension deficit, apraxia and/or visuospatial neglect as assessed through neurological examination, report in the patient’s clinical history or evidence from the neurological examination of behavioral disturbances (i.e. delusions, aggressiveness and severe apathy/depression) that could affect compliance with the rehabilitation programs, presence of non-stabilized fractures, presence of traumatic brain injury, presence of drug resistant epilepsy.

**Fig. 1:**
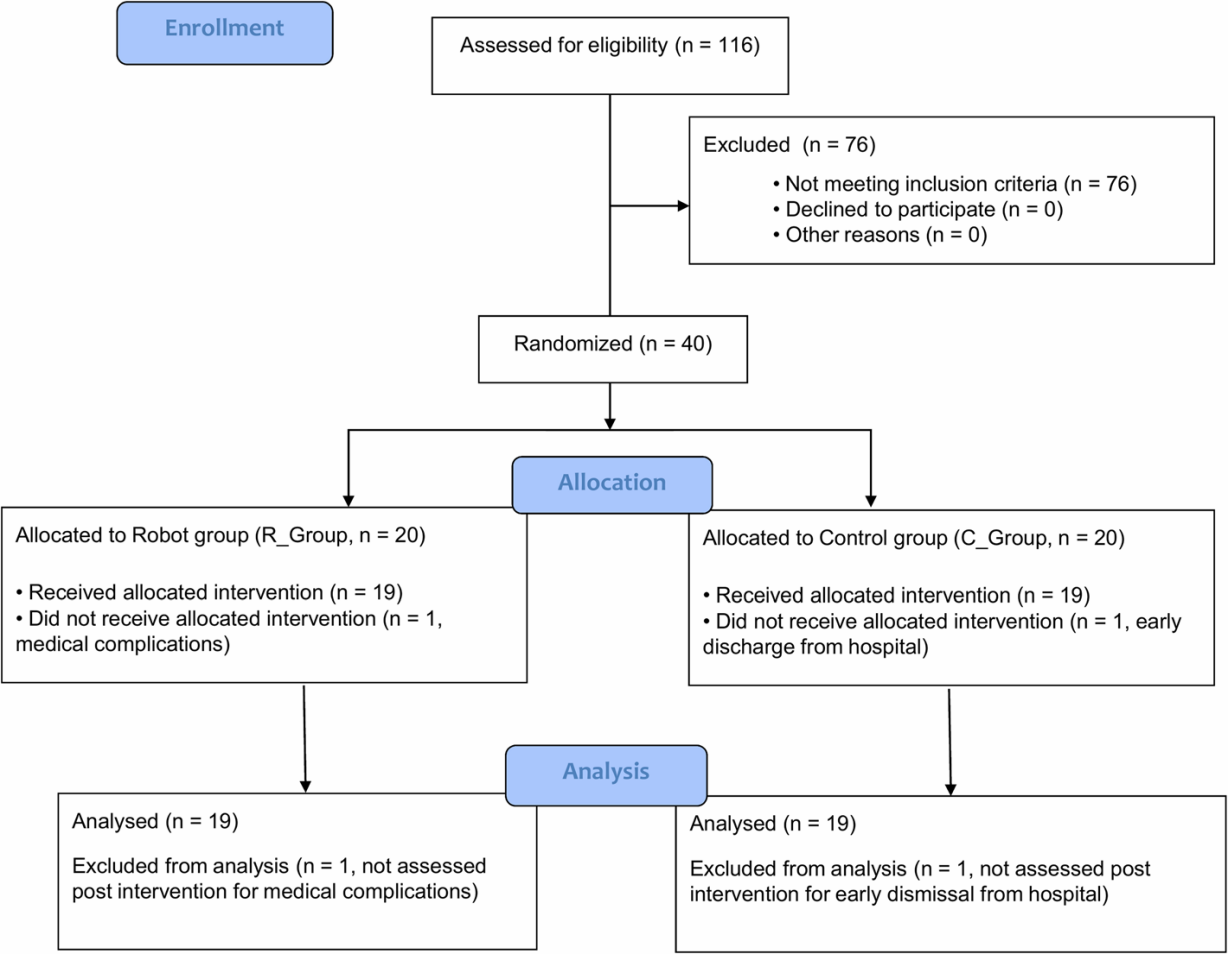
Flow chart of the study

The recruited sample consisted of 40 persons (Fig. 1), in both chronic (> 3 months post stroke) and sub-acute (<= 3 months post stroke) stage post-stroke [61, 62]. Participants were consecutively randomized to the Robot Group (R_Group) or the Control Group (C_Group) using a computerized automated algorithm prepared by an investigator with no clinical role in the study to ensure concealed allocation. Randomization was stratified according to disease onset (<= 3 months or > 3 months) to ensure that the numbers and participants’ chronicity in each group were comparable.

A sample of ten healthy subjects (HS), without any musculoskeletal or neurological disorders, was also recruited to provide normative data related to the instrumented kinematic analysis of the “move-and-place” test (see section Instrumented Assessment – Move-and-place Test).

All participants gave written informed consent to the study that was conformed to the Declaration of Helsinki and was approved by the ethical committee of IRCCS Don Carlo Gnocchi Foundation, Milan, Italy (session October 15, 2014).

### Intervention

Participants in both the Robot and Control groups received a rehabilitation treatment for the affected upper limb consisting of 20 sessions of 45 min each, 5 times a week by trained physiotherapists.

#### Robot group (R_Group)

**Fig. 2:**
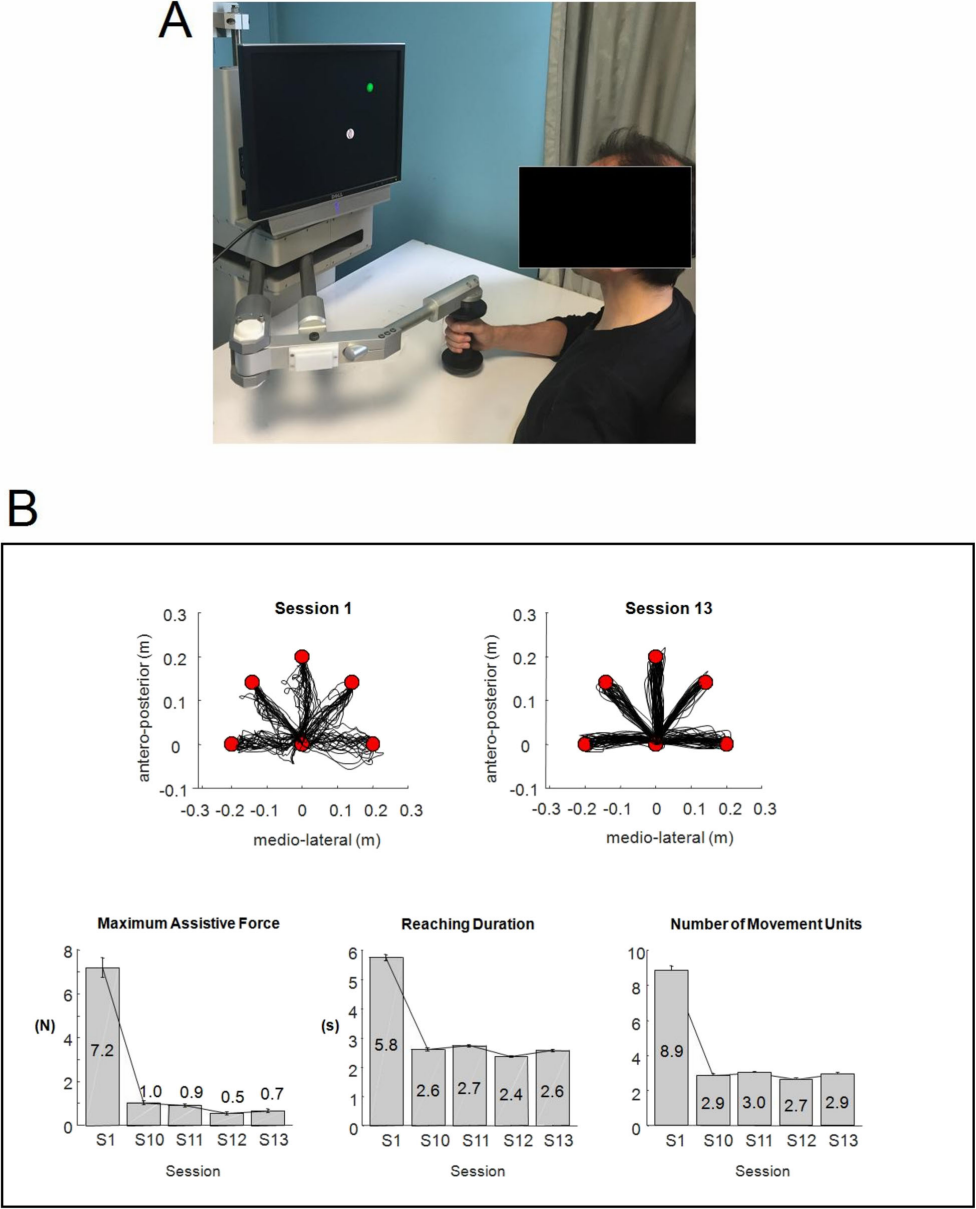
**a** Example of a subject using the robot Braccio di Ferro. **b** Example of a summary report shown to the subject at the end of each training session (in this case the 13th session, S13). The upper panels show the trajectories executed during the first session (S1) and during the session just ended (S13). The lower panels shows the bar plots representing the mean ± standard deviation values of three robot-based indexes (i.e. maximum assistive force generated by the robot, reaching duration and number of movements units) during the first (S1) and the last four sessions (S10 to S13)

Participants allocated to the R_Group received a robot-based training using a planar robotic manipulandum (Braccio di Ferro, Celin s.r.l., Italy [63]) aimed at practicing shoulder and elbow movements in the horizontal plane (Fig. 2a). Subjects were seated on a chair while grasping the handle of the robot with the paretic hand. A large computer screen was used to display the current position of the hand and the target represented by circles with a diameter of 3 cm (Fig. 2a). The task consisted of repeated center-out reaching movements and back, from a central target to a peripheral target randomly presented in one of five positions arranged on a semi-circle with a 20 cm radius (Fig. 2b, upper panels). The robotic system enabled the execution of reaching movements in two force modes, *assist-as-needed* and *resistive*. In the *assist-as-needed mode,* the participant executed the movement while the robot generated a minimally assistive force which helped to reach the target. In particular, after the appearance of the target, no assistive force was delivered for 2 s. At that time, if the participant was not able to reach the target on his/her own, a minimally assistive force was generated by the robot. This force was automatically modulated based on the hand speed: robotic assistance increased if the hand speed decreased below a threshold V1 = 0.03 m/s, decreased if the hand speed grew above a threshold V2 = 0.06 m/s, while it remained constant if the hand speed was between V1 and V2. Maximum generated force was equal to 25 N. This assistance mode enabled the participant to reach the target even in absence of voluntary activity. In the *resistive mode*, the participants executed the reaching movements while the robot generated a spring-like resistive force which opposed hand’s movement. This resistive force was equal to *-K·Δx,* where *K* was the rigidity coefficient and *Δx* was the distance between the current position of the hand and the starting position [64]. The maximum *K* value was 125 N/m, corresponding to a maximum resistive force of 25 N. The implemented robotic paradigm did not provide any constraints (e.g. virtual elastic walls) that prevented the participant from moving away from the straight line between the starting point and the target.

The first session was performed by all participants in the *assist-as-needed* mode. At the beginning of the following sessions, the physiotherapist analyzed the summary report (see the example of Fig. 2b) showing the values of three robot-based indexes (i.e. maximum assistive force, reaching duration and number of movements units) related to the first and the last sessions performed. If the maximum assistive force generated by the robot during the previous session was greater than 1 N, the current session was still executed in the *assist-as-needed* mode, otherwise the physiotherapist changed the exercise to the *resistive* mode, setting the rigidity *K* to the minimum value of 5 N/m. If the participant was unable to reach at least five targets within 10 s each, or if he/she had arm pain, the physiotherapist reloaded the exercise in the *assist-as-needed* mode, otherwise the session was executed in the *resistive* mode. In the subsequent sessions, the value of the rigidity *K* was modulated based on the physiotherapist’s judgment of the summary report related to the previous session (in particular reaching duration and number of movement units).

At the end of each training session, the report was shown also to the participant as a summary feedback about the trend of his/her performances (an example is reported in Fig. 2b).

The number of reaching movements executed during each 45-min session was between 240 in most impaired participants and 500 in less impaired participants. Trunk was not constrained during the training and the training did not directly involve intrinsic movements of the hand.

#### Control group (C_Group)

Participants allocated to the C_Group underwent usual care arm-specific physiotherapy that typically consisted of passive and active mobilization of scapula, shoulder, elbow and wrist, followed by task-oriented exercises that incorporated single or multi-joint movements aimed at improving arm functionality. Task-oriented activities were tailored to participants’ abilities, and included hand to mouth movements, reaching towards and grasping objects, moving objects from one location to another. Participants that were not able to grasp would aim at moving towards objects in various trajectories, pushing them from one setting to another. Progression was obtained by increasing range of motion, number of repetitions and muscular coordination requests. A paper published by Kimberley et al. [65] estimated that a typical number of movements executed in a usual care rehabilitation session, such as that carried out by the C_Group, was around 40–45 repetitions.

### Clinical assessment

Participants were clinically evaluated by a trained examiner, unaware of group assignment, at baseline (T0) and post-training (T1). Clinical assessments included the Fugl-Meyer Motor Assessment of Upper Extremity (FM-UE) [59], the Reaching Performance Scale (RPS) [66] the Modified Ashworth Scale [67], and the Functional Independence Measure (FIM) scale [68].

The FM-UE, is a stroke-specific impairment scale widely accepted as a measure of body function impairment after stroke. Its score ranges from 0 to 66, with higher values indicating lower impairment of the upper limb [59]. The RPS evaluates upper limb motor performance and trunk compensation during reaching to grasp a cardboard cone positioned on a Table 1 cm (close target) and 30 cm (far target) from the edge of the table. The RPS score ranges from 0 to 18, with higher values indicating better performance [66]. The MAS scale rates muscle spasticity from 0 (no increased muscle tone) to 4 (rigid flexion or extension is present) [67]. In the present study MAS was applied to the following muscles of the paretic limb: pectoralis major, biceps brachii, flexors carpi, flexor digitorum profundus, and flexor digitorum superficialis. The FIM scale is a reference standard to measure independence in basic ADLs including self-care, mobility and communication. FIM total score ranges from 18 (maximum level of dependence) to 126 (highest level of independence) [68].

**Table 1:** Demographic and clinical features of Robot group (R_Group) and Control group (C_Group)

Variable	R_Group (*N* = 19)	C_Group (*N* = 19)	*P*-value
	Median (1st-3rd quartile)	Median (1st-3rd quartile)	
Age (years)	67.0 (58.0–70.0)	59.0 (46.0–69.0)	0.234
Time since stroke (months)	7.0 (1.7–11.9)	5.3 (1.9–89.6)	0.797
	Number (%)	Number (%)	
Sex			1.000
Female	9 (47)	9 (47)	
Male	10 (53)	10 (53)	
Stroke Type			0.732
Ischemic	13 (68)	12 (63)	
Hemorrhagic	6 (32)	7 (37)	
Paretic Side			0.511
Right	9 (47)	7 (37)	
Left	10 (53)	12 (63)	
Chronicity			0.511
Chronic	12 (63)	10 (53)	
Sub-acute	7 (37)	9 (47)	

*P*-values indicate the results of Mann-Whitney U Test for age and time since stroke, and of chi-square test for all the other variables

### Instrumented assessment – robot-based indexes

Regarding the R_Group, the following robot-based indexes were computed from the planar reaching trajectories executed during each training session (from 1 to 20): maximum assistive force generated by the robot, mean reaching duration, and number of movement units to reach the target, the latter being a measure of smoothness [32, 33]. The number of movements units was identified by the number of peaks in the velocity profile which met the following criteria: an amplitude greater than 0.02 m/s [32], and a distance in time between two consecutive peaks greater than 0.3 s [40].

### Instrumented assessment – move-and-place test

**Fig. 3:**
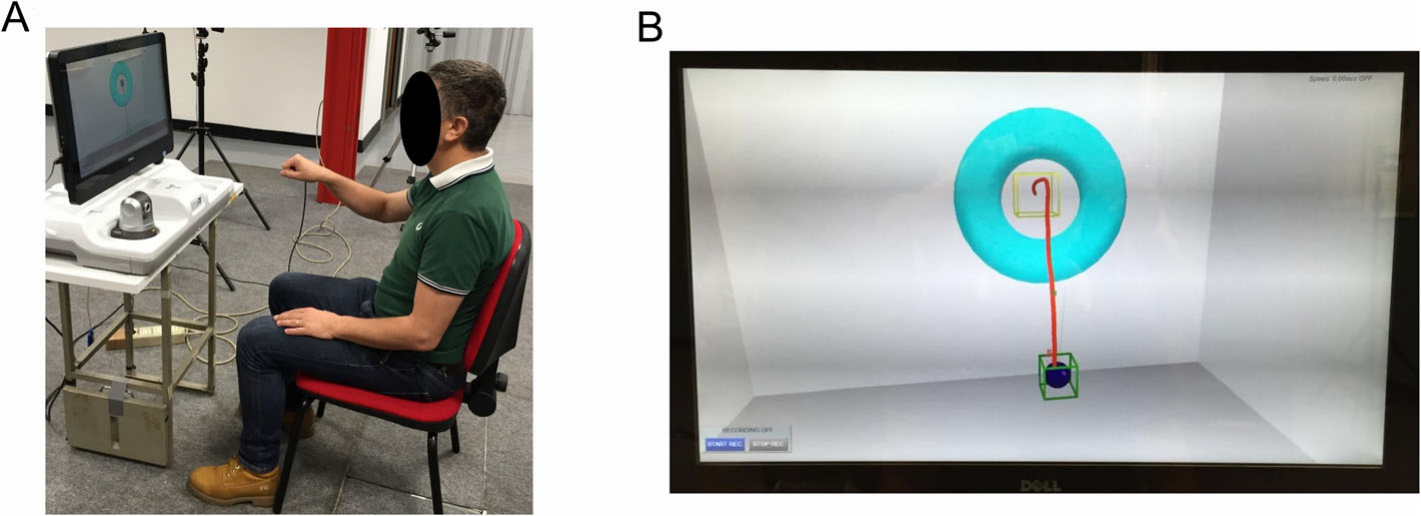
**a** Example of a subject executing the “move-and-place” test using the VRRS virtual reality system. **b** Virtual scenario shown to the subject during the “move-and-place” test. The blue ball represents hand’s movement, the green box is the starting position, the yellow box is the target position. The red line shows the trajectory of one representative subject (not shown during the test)

All participants (R_Group and C_Group) were required to perform an instrumented 3D “move-and-place” test at baseline (T0) and post-training (T1), to assess the effects of rehabilitation on a non-trained functional task. The test was executed using the virtual reality system VRRS® (Khymeia Group Ltd., Italy), as shown in Fig. 3a.

The participant was seated in front of a screen on a chair with a slightly tilted back. At the beginning of the test, the participant was asked to position both hands on thighs, to hold the VRRS electromagnetic sensor with the paretic hand, and to maintain the trunk erect without leaning on the back of the chair. The position of the sensor (i.e. the hand) was represented by a virtual blue ball on the screen. A calibration procedure was performed, using the proprietary software of the VRRS system, so that the starting position of the ball was set inside the green cube (i.e. starting area) displayed on the screen (Fig. 3b). After this procedure, the participant was required to move the virtual ball and place it into the yellow cube (i.e. target area) positioned at an antero-posterior and vertical distance of 36 cm and 26 cm, respectively, from the starting area (Fig. 3b). This 3D virtual task simulated the functional movement of transporting an object onto a shelf. All participants were asked to consecutively repeat the task 12 times. However some of them were not able to perform all repetitions. Similarly not all participants succeeded in completing the task (i.e. place the ball into the target area). A trial was considered valid for the analysis if at least 15% of the antero-posterior and/or vertical trajectory’s component was executed.

The same test was performed by the recruited healthy subjects (HS). Six of them executed the task with the right hand and four with the left hand.

Kinematics of upper limb and trunk were recorded using a 9-camera optoelectronic system (SMART-DX, BTS, Italy) with a sampling frequency of 200 Hz. The system measured the 3D coordinates of nine spherical markers (10 mm diameter) attached to the following body landmarks: C7, manubrium, right and left acromions, lateral humeral condyle, ulnar and radial styloid processes, mid-forearm and hand of the tested limb. Markers’ coordinates were low-pass filtered at 6 Hz and then used to compute trunk, shoulder and elbow angles according to the joint coordinate system method described by Grood and Suntay [69]. Instants of initiation (t_start_) and termination (t_end_) of each movement were computed from the velocity of the hand’s marker. In particular t_start_ was the first frame at which hand’s velocity exceeded 5% of the maximum value, while t_end_ was the first frame at which hand’s velocity fell below the 5% of the maximum value. Hence, the time course of trunk and upper limb angles were time normalized as a percentage of movement duration (t_end_-t_start_). The following outcome measures were then computed from each single repetition and averaged for each participant at both T0 and T1.

- Shoulder/Elbow Coordination Index (unitless): quantified using the cross-correlation analysis at zero time lag between temporal profiles of shoulder and elbow flexion/extension angles [32]. The cross-correlation coefficient ranges from − 1 to 1, with 0 value indicating that the movement of the two joints are completely independent. A high positive coefficient, close to 1, occurs when joint motion is tightly coupled and in the same direction (e.g. shoulder and elbow flex), while a high negative coefficient, close to − 1, indicates that the joint movements are tightly coupled but in opposite directions (e.g. the shoulder flexes and the elbow extends). The latter is the typical condition occurring during the reaching movements in healthy participants.
- Amount of Shoulder Flexion (deg): computed as the shoulder flexion angle at t_end_ with respect to the shoulder angle at the beginning of the movement (t_start_). Increasing values indicate larger amount of flexion.
- Amount of Elbow Extension (deg): computed as the elbow flexion angle at t_end_ with respect to the elbow angle at the beginning of the movement (t_start_). Increasing negative values indicate larger amount of extension.
- Trunk Compensation Index in the sagittal plane (deg): computed following Eq. 1, as the average root-mean-square difference between the mean curve representing the trunk angular movement in the sagittal plane of each participant post-stroke and the normative mean curve representing the same variable in the healthy group. Larger values indicate greater deviation from normal trunk sagittal movement.

1 $$ Trunk\ {Compensation\ Index}_j=\sqrt{\frac{\sum \limits_{i=1}^N{\left({x}_j(i)-{x}_{HS}(i)\right)}^2}{N}} $$

where *j* represents the *j*th participant, *x*_*j*_*(i)* is the trunk angular movement in the sagittal plane of participant *j* at the *i*th time frame, *x*_*HS*_*(i)* is the trunk angular movement in the sagittal plane averaged among the healthy volunteers at the *i*th time frame, and *N* is the number of time frames.

The above instrumented parameters were computed after ensuring that the initial posture (i.e. shoulder, elbow and trunk angles at t = t_start_) was comparable between groups both pre- and post-training and within-group between pre-and post-training (*p* > =0.418).

### Outcome measures

Considering the two aims of the study, two primary outcome measures were chosen, one instrumented and one clinical. The primary instrumented outcome measure was the Shoulder/Elbow Coordination Index. This parameter has previously been applied to the study of reaching movements in healthy subjects and persons post-stroke [32]. Moreover, its psychometric properties have been investigated in a post-stroke population, showing good to excellent reliability [70]. The primary clinical outcome measure was the FM-UE score.

The secondary instrumented outcome measures were the amount of shoulder flexion and elbow extension, and the trunk compensation index. The secondary clinical outcome measures were the proximal and distal portions of the FM-UE (P_FM-UE and D_FM-UE), RPS, MAS of proximal and distal muscles (P_MAS and D_MAS), and FIM. P_MAS and D_MAS scores were computed by summing the MAS scores of the proximal (i.e. pectoralis major and biceps brachii), and distal (i.e. flexors carpi, flexor digitorum profundus, and flexor digitorum superficialis) muscles, respectively.

### Sample size

Considering the first aim of the present study, sample size was estimated using previous published data on the primary instrumented outcome measure (i.e. shoulder/elbow coordination index) [71, 72]. These data showed a post-training mean (standard deviation) change score (positive values indicating improvement) equal to 0.68 (0.69) after robot therapy [71] and equal to − 0.02 (0.16) after a control treatment similar to that administered in the present study [72]. Change scores enabled the computation of the effect size (Cohen’s d = 1.40), which indicated that 24 subjects (12 per group) were necessary to obtain a difference between groups with α = 0.05 and Power (1-β) = 0.9.

### Statistical analysis

#### Baseline assessment

Demographic and clinical baseline scores were compared between the Robot group (R_Group) and the Control group (C_Group) using separate parametric or non-parametric tests based on data distribution and homogeneity of variances (assessed, respectively, with Jarque-Bera test and Levene’s test). Mann-Whitney U Tests were used to compare age and time since stroke, while t-tests for independent samples were used to compare baseline clinical scores (i.e. FM-UE, P_FM_UE, D_FM_UE, RPS, P_MAS, D_MAS, FIM). Chi-square tests were used to compare sex (female/male), stroke type (ischemic/hemorrhagic), paretic side (right/left), and chronicity (chronic/sub-acute). Baseline instrumented parameters (i.e. shoulder/elbow coordination index, amount of shoulder flexion and elbow extension, and trunk compensation index) were compared using ANOVA (analysis of variance) tests with one between-group factor (Group: healthy subjects, R_Group, C_Group) and Bonferroni-Holm post-hoc test. This analysis was performed to assess not only if the baseline instrumented features were comparable between the R_Group and the C_Group, but also to assess if they were significantly different with respect to those describing the healthy group (HS).

#### Robot-based indexes

The robot-based indexes computed from the trajectories executed by the R_Group during the robotic training (i.e. robot-generated force, movement duration and number of movement units) were analyzed using repeated measure ANOVA tests with one within-group factor (Session: 1 to 20).

#### Treatment effect – primary outcome measures

The differential effects of the two treatments were assessed by comparing the change scores (i.e. post-training change from baseline) of the primary outcome measures (i.e. shoulder/elbow coordination index and FM-UE) through ANCOVA (analysis of covariance) tests with one between-group factor (Group: R_Group, C_Group) adjusting for the baseline values of the respective measure. Since the shoulder/elbow coordination data were not normally distributed, a cube transformation (i.e. X^3^) was applied to normalize the distribution. Between-group differences and effect sizes (expressed as Cohen’s *d*) were computed. Cohen’s *d* equal to 0.2, 0.5, and 0.8 represents small, moderate, and large effect sizes, respectively [73]. The statistical analyses were performed following a *per protocol* approach. To verify the results, a supplementary ANCOVA was run including also drop outs with an *intention-to-treat* approach.

#### Treatment effect – secondary outcome measures

Change-scores of the clinical and instrumented secondary outcome measures were compared between groups following the same method used for the primary outcomes (i.e. separate ANCOVA tests with baseline scores as covariates). Moreover, a chi-square test was used to compare the number of clinically improved participants (i.e. post-treatment improvement > = 5 points in FM_UE [74]) between groups. The analyses were performed following a *per-protocol* approach.

#### Correlation analyses

Spearman’s coefficient (ρ) was used to estimate the correlation between instrumented parameters, and between change scores in the instrumented parameters and clinical measures.

#### Ancillary analyses

Further ancillary analyses were performed separately on participants in the sub-acute and chronic stage post-stroke to assess if the two treatments have different effects on these sub-groups. For both sub-groups ANCOVA tests were run to compare the change scores attained by the two treatment groups in all clinical and instrumented measures.

## Results

The flowchart of the study is reported in Fig. 1. Twenty participants post-stroke were allocated to the Robot group (R_Group) and 20 were allocated to the Control group (C_Group). Two persons discontinued the training, one for medical complications unrelated to the study, and one for early discharge from the hospital.

### Baseline assessment

The demographic and clinical features of the participants post-stroke were statistically comparable between the R_Group and the C_Group, as shown in Table 1. Time since stroke of sub-acute participants was between 1 month and 2.7 months in the R_Group, and between 1 month and 2.8 months in the C_Group (*p* = 0.266). Time since stroke of chronic participants was between 4 months and 6 years in the R_Group, and between 5 months and 9 years in the C_Group (*p* = 0.249). The recruited sample of healthy subjects (HS) consisted of 6 females and 4 males with a median age (1st-3rd quartile) of 66.0 (51.0–68.0) years. Sex and age were comparable to those of participants post-stroke (*p* > =0.477).

The baseline values of the clinical and instrumented outcome measures were statistically comparable between the two treatment arms (Table 2). The instrumented parameters related to the R_Group and the C_Group were significantly different with respect to those characterizing the HS group. Participants post-stroke showed lower amount of shoulder flexion and elbow extension. Moreover, the shoulder/elbow coordination index was significantly different from normative data (Table 2). While the HS group showed a mean value close to − 1, which indicated almost perfect counter-phase movements of shoulder and elbow joints, participants post-stroke showed mean values significantly different from − 1, which demonstrated a reduced coordination between shoulder and elbow motion. Finally, the trunk compensation index was significantly larger in both treatment arms compared to the HS group (Table 2), indicating a larger deviation of trunk sagittal movements from normative data. In particular, 29 out of 38 participants post-stroke showed abnormal trunk sagittal movements. Two of them (7%) presented with larger trunk forward rotation, while 27 (93%) presented with abnormal trunk backward rotation.

**Table 2:** Baseline values of clinical and nstrumented outcome measures for healthy subjects (HS_Group) and post-stroke subjects allocated to Robot group (R_Group) and Control group (C_Group)

Outcome measure	HS_Group	R_Group	C_Group	*P*-value
	*(N = 10)* Mean (SD)	*(N = 19)* Mean (SD)	*(N = 19)* Mean (SD)	
Clinical				
FM-UE (0–66)^b^	–	35.3 (18.6)	28.1 (18.5)	0.238
P_FM-UE (0–42)^b^	–	23.6 (9.7)	19.6 (11.2)	0.253
D_FM-UE (0–24)^b^	–	11.7 (9.2)	8.4 (7.8)	0.244
RPS (0–36) ^b^	–	18.9 (12.9)	12.4 (14.8)	0.156
P_MAS (0–8) ^c^	–	1.9 (1.8)	2.3 (1.3)	0.229
D_MAS (0–12) ^c^	–	2.8 (3.1)	3.0 (2.6)	0.516
FIM (18–126) ^b^	–	99.9 (14.1)	92.0 (16.7)	0.124
Instrumental				
Shoulder/Elbow Coordination Index (unitless) ^c^	−0.92 (0.05)	−0.31 (0.65)^a^	−0.33 (0.62)^a^	< 0.001
Amount of Shoulder Flexion (deg) ^b^	73.0 (9.3)	34.1 (19.8)^a^	27.0 (29.0)^a^	< 0.001
Amount of Elbow Extension (deg) ^c^	−58.3 (11.8)	−12.7 (30.5)^a^	−17.9 (35.2)^a^	< 0.001
Trunk Compensation Index – Sagittal Plane (deg) ^c^	3.3 (1.8)	9.3 (4.5)^a^	9.2 (6.2)^a^	0.006

*SD* standard deviation, *FM-UE* Fugl-Meyer Motor Assessment for the Upper Extremities, *P_FM-UE and D_FM-UE* proximal and distal portion of FM-UE, *RPS* Reaching Performance Scale, *MAS* Modified Ashworth Scale, *P_MAS and D_MAS* MAS for proximal and distal muscles, *FIM* Functional Independence Measure. *P*-values indicate the results of the comparison between R_Group and C_Group (independent sample t-test) and among HS_Group, R_Group and C_Group (one-way ANOVA).

^a^statistically significant different with respect to HS_Group (Bonferroni-Holm post hoc test)

^b^Higher scores indicate better performance

^c^Lower scores indicate better performance

### Robot-based indexes

**Fig. 4:**
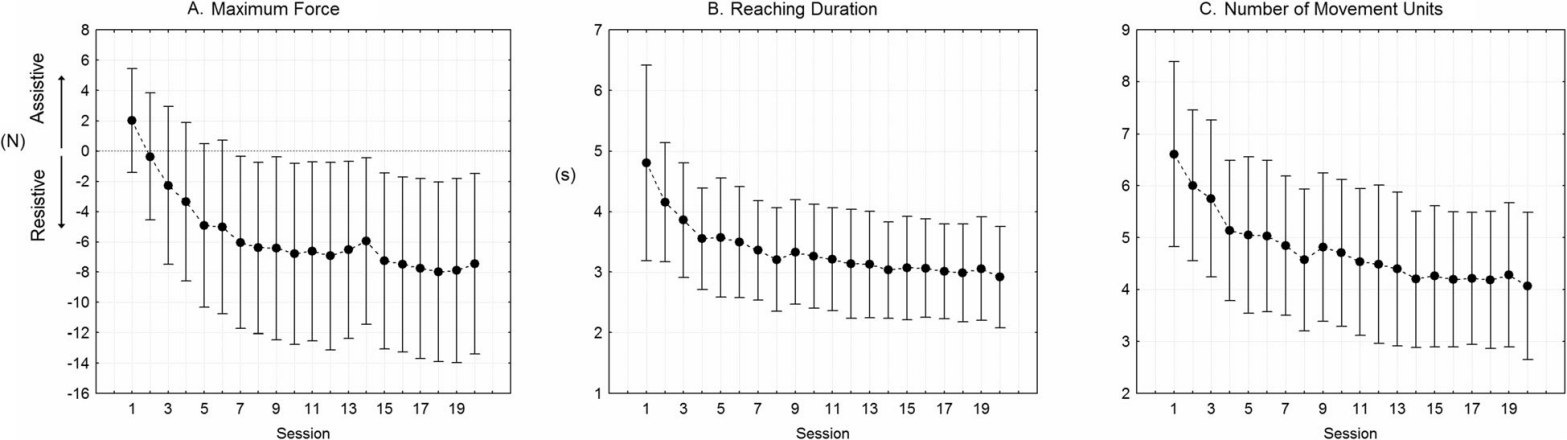
Robot-based indexes computed from the reaching trajectories executed during the 20 sessions of robot therapy. Point: mean; whisker: 95% confidence interval. **a** Maximum force generated by the robot. Positive and negative values indicate, respectively, assistive and resistive force. **b** Mean duration of reaching movements. **c** Number of movement units. Lower values indicate smoother movements

The robot-based indexes related to the 20-session robotic training executed by the R_Group revealed a gradual improvement of the performances, as shown in Fig. 4. The maximum robot-generated force significantly decreased during the training (F_19,361_ = 16.72, *p* < 0.001), moving from positive values (i.e. assistive force) to negative values (i.e. resistive force) (Fig. 4a). The mean reaching duration decreased significantly (F_19,361_ = 8.94, p < 0.001) (Fig. 4b), as well as the number of movement units to reach the target (F_19,361_ = 13.21, *p* < 0.001) (Fig. 4c).

### Treatment effect: instrumented assessment

Regarding the change score in the primary instrumented outcome measure (i.e. the shoulder/elbow coordination index), a statistically significant difference was found between groups (F_1,35_ = 6.04, *p* = 0.019), with the R_Group showing a larger improvement of the inter-joint coordination, as demonstrated by the larger decrease of the index towards normative values (Table 3). The effect size favoring the R_Group was large (Cohen’s *d* = 0.82). The *Intention-to-treat* analysis confirmed this result, showing a larger improvement of the shoulder/elbow coordination index in the R_Group (R_Group: − 0.38 ± 0.57; C_Group: − 0.04 ± 0.13, F_1,37_ = 7.11, *p* = 0.011).

**Table 3:** Change scores (post – baseline values) of instrumented outcome measures for Robot group (R_Group) and Control group (C_Group)

Outcome measure	R_Group	C_Group	Between-group difference	*P*-value	Cohen’s *d*
	*(N = 19)* Mean (SD)	*(N = 19)* Mean (SD)	*(R_Group-C_Group)*^*a*^ Mean (95% CI)		Mean (95% CI)
Primary					
Shoulder/Elbow Coordination Index (unitless) ^c^	−0.38 (0.57)	−0.04 (0.13)	− 0.14 (− 0.25 to − 0.03)	0.019	−0.82 (−1.48 to − 0.16)
Secondary					
Amount of Shoulder Flexion (deg) ^b^	10.9 (12.5)	7.6 (15.9)	4.7 (−4.3 to 13.8)	0.297	0.36 (−0.29 to 1.00)
Amount of Elbow Extension (deg) ^c^	−25.8 (35.1)	−6.1 (19.5)	−17.4 (−33.8 to −0.98)	0.038	−0.72 (− 1.37 to − 0.06)
Trunk Compensation Index – Sagittal Plane (deg) ^c^	−4.01 (5.10)	0.69 (5.72)	−4.63 (−7.41 to − 1.84)	0.002	−1.12 (− 1.81 to − 0.44)

*SD* standard deviation, *95% CI* 95% Confidence Interval. *P*-values indicate the results of the comparison between R_Group and C_Group (analysis of covariance, ANCOVA)

^a^Adjusted for baseline score by ANCOVA

^b^Higher scores indicate better performance

^c^Lower scores indicate better performance

As for the secondary instrumented outcome measures, the R_Group attained a greater post-training increase in the amount of elbow extension (F_1,35_ = 4.63, *p* = 0.038) and a larger decrease of the trunk compensation in the sagittal plane (F_1,35_ = 11.38, *p* = 0.002) (see Table 3). The effect size favoring the R_Group was moderate for the elbow extension (Cohen’s *d* = − 0.72) and large for the trunk sagittal compensation (Cohen’s *d* = − 1.12). By contrast, the increase in the amount of shoulder flexion was comparable between groups (F_1,35_ = 1.12, *p* = 0.297).

**Fig. 5:**
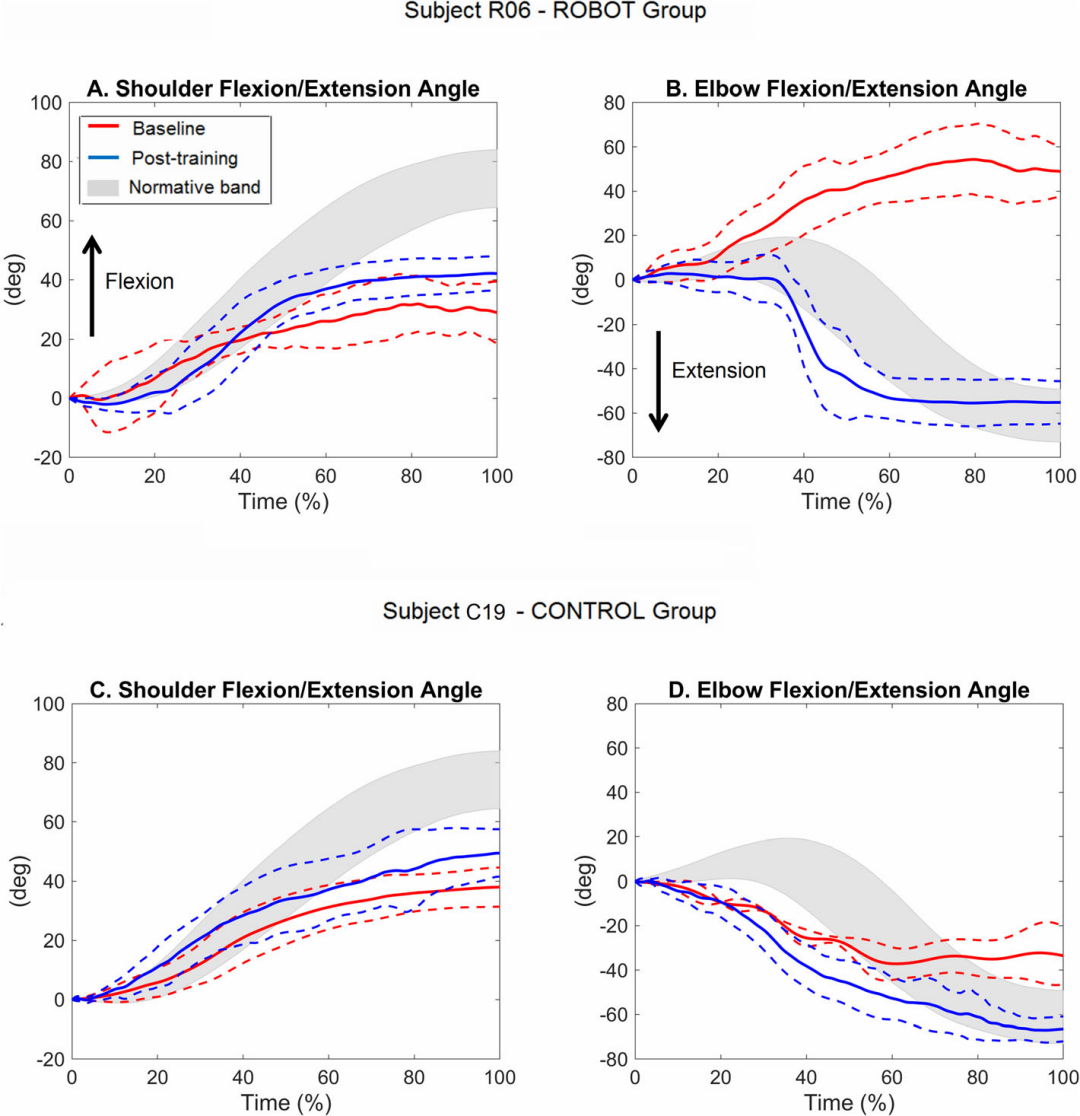
Example of temporal profiles (within-subject mean ± standard deviation curves) of shoulder (**a**, **c**) and elbow (**b**, **d**) flexion/extension angles during the “move-and-place” test executed pre- (red lines) and post-training (blue lines) by two participants post-stroke from the ROBOT group (participant R06, upper panels) and the CONTROL group (participant C19, lower panels). Gray bands represent the healthy subjects mean ± standard deviation curve. All curves are reported after subtraction of the initial values

Examples of the temporal profiles of shoulder flexion and elbow extension angles during the “move-and-place” task are shown in Fig. 5. The kinematic signals are related to the pre- and post-training performances of two participants post-stroke (R06 and C19) with comparable FM-UE baseline scores (R06: 32 points, C19: 35 points). It can be noticed that both participants attained a post-training increase towards normative values of the amount of shoulder flexion (Fig. 5a,c) and elbow extension (Fig. 5b,d). In particular, from Fig. 5b it can be noticed that the participant R06 at baseline abnormally flexed the elbow instead of extending it (red line), while after the robotic treatment he/she recovered an almost normal pattern of elbow extension (blue line).

**Fig. 6:**
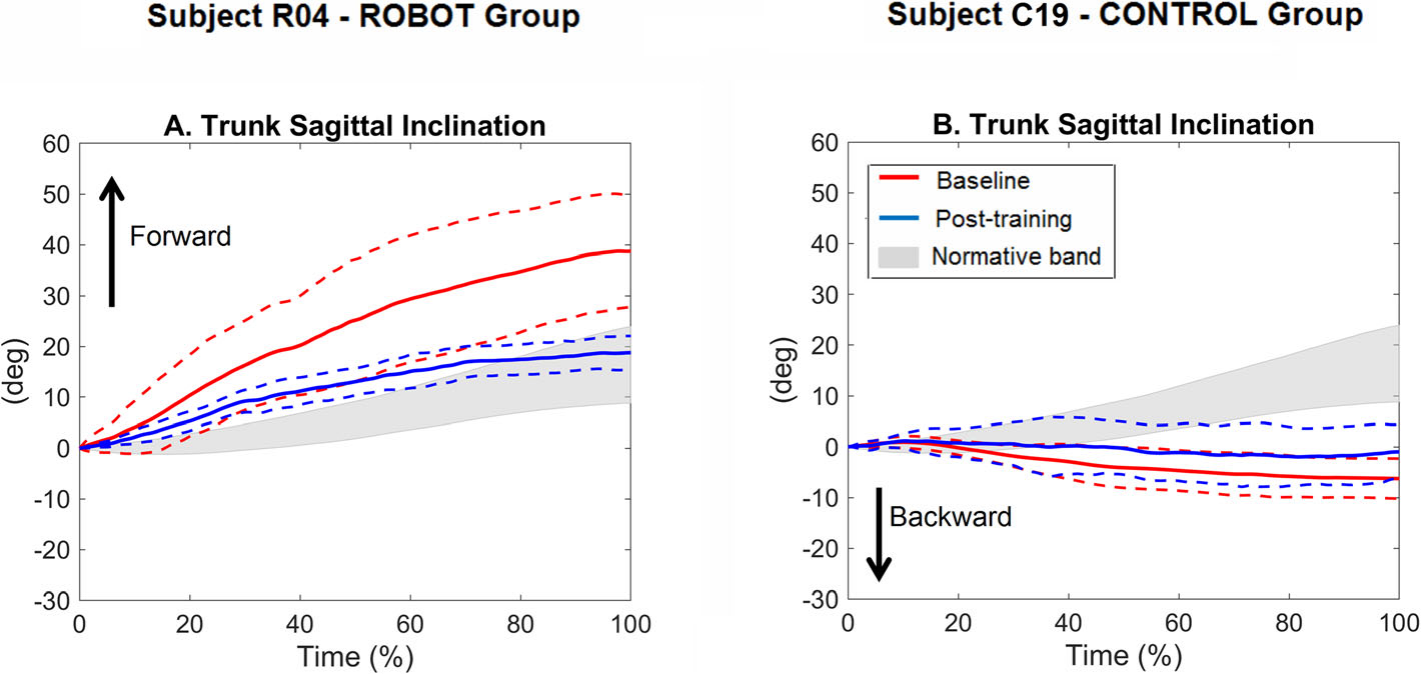
Example of temporal profiles (within-subject mean ± standard deviation curves) of the trunk sagittal inclination during the “move-and-place” test executed pre- (red lines) and post-training (blue lines) by two participants post-stroke from the ROBOT group (participant R04, **a**) and the CONTROL group (participant C19, **b**). Gray bands represent the healthy subjects mean ± standard deviation curve. All curves are reported after subtraction of the initial values

Examples of the temporal profiles of trunk inclination in the sagittal plane are depicted in Fig. 6. The angles referred to two participants post-stroke (R04 and C19) with comparable FM_UE baseline scores (R04: 31 points; C19: 35 points). It can be noticed that at baseline (red lines) both participants presented with abnormal trunk sagittal movement compared to normative data. In particular R04 showed larger forward bending (Fig. 6a), while C19 showed larger backward inclination (Fig. 6b). Both participants, in particular R04, reduced these compensatory movements after the training (blue lines), thus approaching the normative curve.

### Treatment effect: clinical assessment

Regarding the clinical change scores, both groups attained a clinically significant mean improvement of the FM-UE score (> = 5 points) (see Table 4). The improvement was comparable between treatment arms (F_1,35_ = 1.06, *p* = 0.311). Inclusion of drop outs (*intention-to-treat* analysis) confirmed the above results, showing comparable change-scores in the two groups (R_Group: 6.7 ± 6.3; C_Group: 5.6 ± 9.2, F_1,37_ = 1.21, *p* = 0.278).

**Table 4:** Change scores (post-training change from baseline) of clinical outcome measures for Robot group (R_Group) and Control group (C_Group)

Outcome measure	R_Group	C_Group	Between-group difference	*P*-value	Cohen’s *d*
	*(N = 19)* Mean (SD)	*(N = 19)* Mean (SD)	*(R_Group-C_Group)*^*a*^ Mean (95% CI)		Mean (95% CI)
Primary					
FM-UE (0–66)^b^	7.0 (6.3)	6.2 (9.3)	2.4 (−2.3 to 7.1)	0.311	0.35 (−0.29 to 0.99)
Secondary					
P_FM-UE (0–42)^b^	3.6 (4.3)	3.7 (5.7)	1.6 (−1.8 to 5.0)	0.339	0.33 (−0.31 to 0.97)
D_FM-UE (0–24) ^b^	2.7 (3.6)	2.4 (4.5)	1.0 (−1.5 to 3.5)	0.441	0.26 (−0.38 to 0.90)
RPS (0–36) ^b^	4.1 (5.2)	3.2 (8.1)	2.1 (−2.2 to 6.4)	0.328	0.34 (−0.31 to 0.98)
P_MAS (0–8) ^c^	−0.5 (1.3)	0.2 (1.2)	−0.9 (−1.6 to − 0.2)	0.018	−0.83 (− 1.49 to − 0.17)
D_MAS (0–12) ^c^	−0.1 (2.1)	0.2 (1.7)	−0.4 (− 1.5 to 0.7)	0.499	− 0.22 (− 0.87 to 0.41)
FIM (18–126) ^b^	9.3 (5.8)	8.7 (11.6)	2.3 (−3.6 to 8.2)	0.439	0.27 (− 0.37 to 0.90)

*SD* standard deviation, *95% CI* 95% Confidence Interval, *FM_UE* Fugl-Meyer motor assessment for the Upper Extremities, *P_FM-UE and D_FM-UE* proximal and distal portion of FM-UE, *RPS* Reaching Performance Scale, *MAS* Modified Ashworth Scale, *P_MAS and D_MAS* MAS for proximal and distal muscles, *FIM* Functional Independence Measure. *P*-values indicate the results of the comparison between R_Group and C_Group (analysis of covariance, ANCOVA)

^a^Adjusted for baseline score by ANCOVA

^b^Higher scores indicate better performance

^c^Lower scores indicate better performance

Regarding the secondary clinical outcome measures, change scores were comparable between groups (Table 4), with the exception of MAS score of proximal muscles (P_MAS) (F_1,35_ = 6.16, *p* = 0.018). In particular this score decreased post-training in the R_Group while it slightly increased in the C_Group, showing that spasticity of proximal muscles was reduced in the R_Group only, with a large effect size (Cohen’s *d* = − 0.83). Finally, the number of participants who reached a clinically significant improvement in the FM-UE (> = 5 points) was comparable between groups (R_Group: 12 out of 19 subjects; C_Group: 9 out of 19 subjects, *p* = 0.328).

### Correlation analyses

Statistically significant correlations were found between the trunk compensation index and the shoulder/elbow coordination index (*ρ* = 0.43, *p* < 0.001), the amount of shoulder flexion (*ρ* = − 0.50, *p* < 0.001) and the amount of elbow extension (ρ = 0.44, *p* < 0.001). The same analysis, performed on those participants showing abnormal trunk backward rotation at baseline (*n* = 27), revealed that the trunk compensation index significantly correlated with the amount of shoulder flexion (*ρ* = − 0.50, *p* = 0.007) but not with the amount of elbow extension (*ρ* = 0.31, *p* = 0.109).

The change-score in the primary instrumented outcome measure (i.e. shoulder/elbow coordination index) did not correlate with the change-score in the primary clinical outcome measure (i.e. FM-UE score) neither in the R_Group (ρ = − 0.22, *p* = 0.361) nor in the C_Group C (ρ = 0.10, *p* = 0.694), while it correlated significantly with the change-score in the proximal portion of the FM-UE (P_FM-UE) in the R_Group only (R_Group: ρ = − 0.48, *p* = 0.038; C_Group: ρ = 0.16, *p* = 0.526).

### Ancillary analyses

**Fig. 7:**
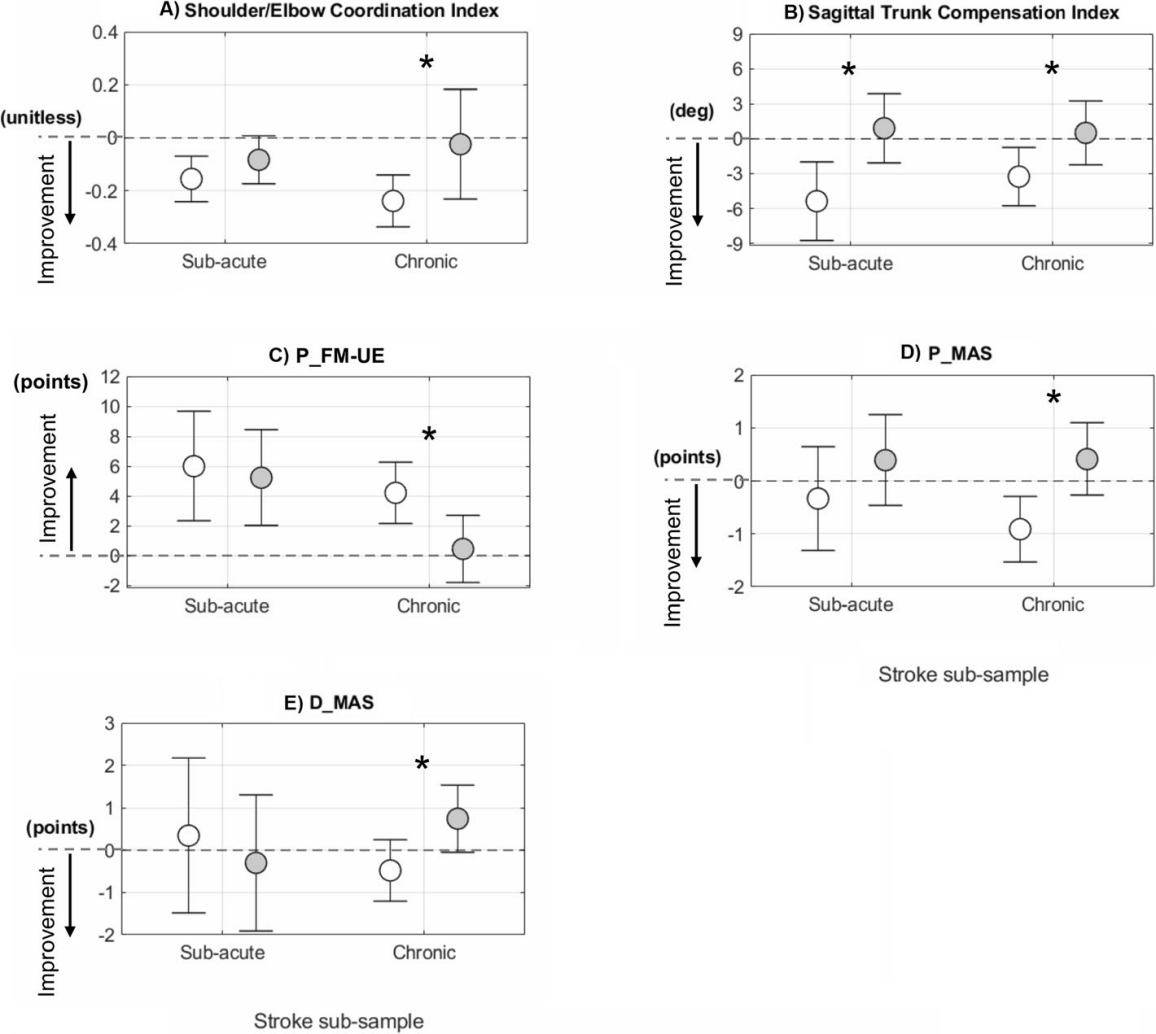
Post-training change scores from baseline attained by sub-acute and chronic participants post-stroke after robot therapy (R, white circles) and control intervention (C, gray circles). Circles and whiskers represent, respectively, mean change score and 95% confidence interval adjusted for baseline score through ANCOVA procedure. * *p* < 0.05 (R versus C, ANCOVA test). P_FM-UE: proximal portion of Fugl-Meyer motor assessment for the Upper Extremities; P_MAS: Modified Ashworth Scale for proximal muscles; D_MAS: Modified Ashworth Scale for distal muscles

The ancillary analysis of the instrumented parameters describing the participants in the sub-acute stage post-stroke showed comparable effects of the two interventions, with the exception of the trunk compensation index which decreased significantly more in the R_Group (F_1,13_ = 9.02, *p* = 0.010) (Fig. 7b). The same analysis on the participants in the chronic stage confirmed this result (F_1,19_ = 4.47, *p* = 0.048) (Fig. 7b) and revealed a significantly larger increase of shoulder/elbow coordination in the R_Group (F_1,19_ = 5.26, *p* = 0.033) (Fig. 7a).

The ancillary analysis of the clinical measures revealed that the two interventions had similar effects on the FM-UE in both sub-acute (change score R_Group: 8.1 ± 7.8; C_Group; 9.8 ± 9.9; F_1,13_ = 0.71; *p* = 0.414, Cohen’s *d* = 0.47) and chronic participants post-stroke (R_Group: 6.3 ± 5.4; C_Group: 2.9 ± 7.8; F_1,19_ = 6.64, *p* = 0.137; Cohen’s *d* = 0.70). As for the secondary clinical outcome measures, no statistically significant difference between the two interventions was found in the sub-acute sample (Fig. 7c-e), while a significantly larger effects of robot therapy compared to control intervention was found in chronic participants in the proximal portion of the FM-UE and in MAS score of proximal and distal muscles (Fig. 7c-e).

## Discussion

The present study compared the effects of a planar robotic training versus arm-specific physiotherapy on (1) upper body kinematics and (2) arm function in persons post-stroke. The analysis of the primary outcome measures showed that, compared to the control intervention, the robotic training induced a larger improvement in the coordination between shoulder and elbow joints, and a comparable amelioration of the arm function as measured by the FM-UE. The analysis of the secondary outcome measures revealed, in the R_Group, a larger improvement of upper body kinematics during a non trained 3D functional task, and a greater reduction in spasticity of proximal muscles. The positive effects of the robotic rehabilitation seemed more pronounced in the subsample of chronic participants.

Both interventions were well accepted by the participants and no adverse events were observed in either cases.

### Robot-based instrumented parameters

The robot-based indexes related to the R_Group showed a gradual and significant decrease of robot-generated forces, which passed, across sessions, from positive values, meaning assistance from the robot, to negative values, meaning resistance from the robot. Despite this gradual increase of exercise difficulty, the planar reaching movements became faster and smoother across sessions, confirming previous findings about the ability of persons post-stroke to improve the execution of intensively practiced tasks [28, 40]. The progressive improvement of smoothness during the robotic training seemed particularly interesting since previous literature has shown that the segmented structure typical of arm movements in persons post-stroke can be attributable to a reduced inter-joint coordination [34]. In this context, the gradually increase of smoothness across the robot-based sessions represented an indirect indication of improved coordinative processes during planar reaching movements [40, 50, 75]. Moreover, the smoothness increase could also be due to the sub-movements temporal blending underlying post-stroke recovery, as suggested by Rohrer et al. [75, 76].

### Effects of robot therapy versus arm-specific physiotherapy on motor control strategies, as measured by instrumented kinematic analysis

More interesting findings emerged from the kinematic analysis of the “move-and-place” task. The baseline assessment showed that the participants post-stroke executed the task with a significant impairment of shoulder/elbow coordination (i.e. the primary outcome) that was accompanied by a statistically significant reduction of the amount of shoulder flexion and elbow extension, as found in previous studies [30, 32–34, 37]. In addition, the results showed also abnormal compensatory sagittal movements of the trunk in 76% of the participants post-stroke. Interestingly, most of these participants (93%) presented with an abnormal trunk backward rotation, rather than a larger forward bending that is more commonly adopted during horizontal tasks to overcome the limited reaching distance of the arm [30, 32, 33, 35, 37]. Since the trunk backward rotation significantly correlated with the amount of shoulder flexion (ρ = − 0.50, *p* = 0.008) and not with the amount of elbow extension (ρ = 0.32, *p* = 0.109), it can be suggested that this type of trunk compensation was specifically associated with the impairment of shoulder flexion which plays a primary role in vertical movements typical of 3D tasks.

The analysis of the differential effects of the two treatments revealed that the R_Group attained significantly larger improvements than the C_Group in the “move-and-place” task that was executed outside the robotic workspace and that required vertical arm movements not specifically practiced during the robotic training. These findings enforced and complemented previous published results about the transfer (at least in the short-term) of planar robot therapy effects to untrained tasks requiring movements in the horizontal plane only [28, 53, 55].

Specifically, the present results showed that, compared to the C_Group, the R_Group attained a significantly larger improvement of the shoulder/elbow coordination that was accompanied by a larger increase in the amount of elbow extension. These findings may be explained by a number of factors. First, the robotic training was significantly more intensive than the control treatment. Indeed, the possibility of administering more movements during the same time interval is a hallmark of robot-therapy [16] and an advantage over the “usual care” arm-specific physiotherapy chosen as control intervention, even though the latter had the advantage of including various functional movements more similar to ADLs [77]. Second, the greater improvements in the R_Group can be attributed to the application of other three principles of motor learning in addition to the high training intensity. In particular, the robotic paradigm applied in the present study (i) was highly specific in practicing shoulder and elbow coordinated motions during different target-directed movements (i.e. reaching virtual targets placed in different directions) [77, 78], (ii) promoted the active participation of the subject also in the case of severely impaired persons, who were able to perform the task by exploiting their minimum residual activity through the assist-as-needed interaction with the robot [79]. This, in turn, contributed also to increase their motivation [79], and (iii) provided participants with both online visual and haptic feedback and quantitative summary feedback about their performances [26]. A third explanation of the present findings could be that during the planar robotic training the arm weight was supported. Previous studies have highlighted the beneficial effects of the arm-weight support on motor control of the upper limb [80, 81]. In particular, arm-weight support has been demonstrated to reduce the unwanted coupling between shoulder and elbow typical of persons post-stroke [80, 82], and to facilitate the active movements of the arm by reducing the activity of muscles involved in reaching, in particular those counteracting the gravity [81, 83, 84]. Finally, a trend favoring the R_Group was present at baseline in terms of upper limb impairment. This may have influenced the results. However, we think that this hypothesis can be excluded given the lack of correlation between the FM-UE baseline scores and the change scores in the instrumented measures (0.02 < = ρ < =0.10, p > = 0.562), suggesting that the improvements of arm kinematics were independent from the baseline level of impairment.

Interestingly, although the trunk was not constrained during the training (as described by Michaelson et al. [85]) a significantly higher reduction of trunk sagittal compensation was found in the R_Group compared to the C_Group, in accordance with Hsieh et al. [56]. This result is probably due to the larger improvements in upper limb kinematics attained by the R_Group. In fact, the correlation analysis between the instrumented parameters showed that better movements of the upper limb were associated with less compensation of the trunk, as previously found also by Cirstea and Levin [37].

Taken together all the above results indicated that the motor control strategies adopted to accomplish the “move-and-place” task improved significantly more after robot therapy than after control intervention. It can be speculated, as discussed in previous studies [27, 30], that the proposed planar robotic training enhanced neural plasticity [21, 26] and induced cortical reorganization supporting true recovery (i.e. the person partly regained the ability to accomplish the task in a way more similar to healthy subjects) rather than compensation (i.e. the person executed the task using abnormal trunk movements) [50]. Further investigations including functional imaging studies and follow-up assessments are warranted to test this hypothesis [86].

### Effects of robot therapy versus arm-specific physiotherapy on arm function, basic ADL and muscle spasticity, as measured by clinical scales

The analysis of the clinical outcome measures showed that FM_UE change scores were comparable between intervention arms, with mean values being above the clinically significant threshold of 5 points in both groups (R_Group: 7 points; C_Group: 6 points). Nonetheless a higher percentage of subjects in the R_Group (63%) attained a clinically significant improvement of the FM-UE compared to the C_Group (47%). This was reflected in an effect size in favor of the R_Group (Cohen’s d = 0.35) although the difference was not statistically significant. Noteworthy, this FM-UE effect size was comparable or superior to values reported by recent reviews on robot therapy versus physiotherapy without technological devices (Cohen’s d between 0.12 and 0.39 [16, 17, 87]). The result was confirmed considering both dose-matched (Cohen’s d = 0.23 [16]) and non-dose-matched trials (Cohen’s d = 0.08 [16]). In addition, the adjusted mean difference between groups in FM_UE (2.4 points) was similar to that found in a very recent study involving 770 post-stroke participants (2.79 points) [88]. Since these reviews and studies analyzed a larger number of subjects (from 228 [16] to 1452 [17]), the lack of a statistically significant difference found in the present study may be ascribed to the small sample size. Similar results were seen on secondary measures FIM and RPS that showed small effect sizes favoring the R_Group (0.27 and 0.34 respectively) without the difference being statistically significant when adjusted for by baseline scores.

When proximal and distal components of the FM-UE scale (P_FM-UE and D_FM-UE) were separated, the improvements were similar and non significantly different between groups. However, a statistically significant correlation between the change-scores in the shoulder/elbow coordination index and in the P_FM-UE was found in the R_Group only, suggesting that the reduction of proximal arm impairment in this group was mainly due to the improvement of inter-joint coordination intensively practiced during the robotic training. In addition, the analysis of MAS scores representing spasticity, revealed significantly larger improvements in the proximal muscles in the R_Group while distally the two groups remained similar. This reduction in spasticity of proximal muscles following robot therapy was close to the clinically significant threshold of − 1 point [89] and was in contrast with other studies showing overall comparable effects of robot therapy and traditional physiotherapy in reducing muscle tone [16, 90, 91]. It is possible that the high intensity of the proposed robotic paradigm aimed at practicing shoulder and elbow movements was effective in reducing spasticity only of the muscles directly involved in the trained task.

### Effects of robot therapy versus arm-specific physiotherapy on sub-acute and chronic participants post-stroke

Even though caution must be taken given the small sample sizes, the secondary ancillary analyses performed separately on sub-acute and chronic participants post-stroke indicated different effects of the robotic intervention in the two sub-groups.

In the sub-acute participants, the two interventions had similar effects on all outcome measures. These results seemed in contrast with the systematic review of Mehrholz et al. [17] showing larger improvements after robot-therapy in sub-acute participants [17]. However, the present findings may be ascribed to the small sample analyzed (7 and 9 participants in R_Group and C_Group, respectively), and also to the high variability in the upper limb function of these participants at baseline. In particular, their pre-treatment FM-UE scores ranges from 7 to 61 points, indicating that the included sub-acute participants were not prognostically comparable [16, 92]. To address this issue, future studies should focus on more homogeneous sub-acute populations, also taking into account the role of novel neurophysiologic biomarkers, such as the response to transcranial magnetic stimulation, potentially able to predict the effects of arm rehabilitation for each participant [93].

Different results were found after the analysis of chronic participants. The instrumented kinematic analysis showed that the robotic training induced a significantly larger post-training improvement of the shoulder/elbow coordination that was accompanied by a significantly higher reduction of trunk sagittal compensation during the “move-and-place” test. These results, in turn, suggested a transfer of robot training effects to a non trained task also in chronic participants. Regarding clinical scales, the two interventions had comparable effects on the FM-UE, while the robotic training induced larger reduction of proximal arm impairment (P_FM-UE) and muscle tone. Interestingly the robotic training induced an improvement in the FM-UE (6.2 points) that was comparable to that obtained by chronic participants treated with more intensive upper limb rehabilitation programs (8 points [78] and 6 points [94]). These findings confirmed previous results indicating that robotic rehabilitation may be more effective than conventional treatments for chronic participants [16, 90]. Moreover, they supported the notion that cortical reorganization is present also in the chronic stages post-stroke [86] and can be enhanced by high-intensity treatments [94–96].

### Added values of instrumented analysis

The present findings highlighted the high sensitivity of the instrumented kinematic assessment in detecting differences in upper body movements that are not captured by the clinical scales [31]. The instrumented analysis here applied enabled the quantification of movement quality both in trained movements and in a non-trained task, thus providing information about the transfer of treatment’s effects to untrained activities, at least in the short-term. Importantly, the kinematic analysis enabled the distinction between “recovery” and “compensation” that is an aspect of paramount importance in rehabilitation. The rehabilitative treatments should be primarily aimed at improving arm function by restoring a more physiological movement pattern [31, 34]. However, in severely impaired persons, the interventions may be more focused on improving ADLs through the development of better compensatory strategies. In this context, instrumented analysis could contribute in deciding the most appropriate rehabilitation approach. This further highlighted the importance of combining instrumented evaluations and standard clinical assessments.

### Study limitations

Some limitations should be considered in the present study. First, the size of the examined sample was dimensioned only on the instrumented primary outcome measure and should be increased to detect a difference in the primary clinical outcome measure. An a posteriori power analysis on FM-UE change score showed that 130 subjects per group are required to achieve a between-group effect size of 0.35, given α = 0.05 and 1-β = 0.8. A second limitation is the lack of follow-up assessments that did not allow the analysis of retention of training effects. Future studies on a larger sample, including also follow-up assessments, should be performed to corroborate present findings and assess long-term training effects. Third, the administered robotic exercise was based on a simple virtual scenario that was maintained fixed across all sessions. Possibly, the implementation of more sophisticated computer-controlled environments enabling more challenging tasks in the form of games would increase participants’ motivation and interaction with the environment thus increasing the clinical effects of the robotic training [15, 26, 97, 98]. Finally, the inclusion of distal robotic components [98, 99] should also be addressed to possibly enhance the effect on wrist and hand and promote the transfer to ADLs.

## Conclusion

Intensive planar robotic rehabilitation aimed at practicing shoulder and elbow movements was more effective than arm-specific physiotherapy in improving arm inter-joint coordination in persons post-stroke, and was as effective as arm-specific physiotherapy in reducing upper limb impairment as measured by the FM-UE. The instrumented kinematic analysis of upper limb and trunk showed that the robotic training induced larger improvements in the motor control strategies adopted to perform an untrained functional task involving also vertical movements against gravity not directly practiced during the training. Future studies should be performed to assess if the use of exoskeleton systems (e.g. [93]) enabling the execution of 3D movements may further enhance the transfer of the rehabilitation effects to untrained ADLs.

## Data Availability

The dataset used and/or analyzed during the current study is available from the corresponding author on reasonable request.

## Abbreviations

ADL: Activities of daily living
C_Group: Control Group
D_FM-UE: Distal portion of the Fugl-Meyer Motor Assessment of Upper Extremity
D_MAS: Modified Ashworth Scale of distal muscles
FIM: Functional Independence Measure
FM-UE: Fugl-Meyer Motor Assessment of Upper Extremity
HS: Healthy subjects
IT – NIHSS: Italian version of the National Institute of Health stroke scale
MAS: Modified Ashworth Scale
P_FM-UE: Proximal portion of the Fugl-Meyer Motor Assessment of Upper Extremity
P_MAS: Modified Ashworth Scale of proximal muscles
R_Group: Robot Group
RPS: Reaching Performance Scale
